# Genetic Structure of Native Blue Honeysuckle Populations in the Western and Eastern Eurasian Ranges

**DOI:** 10.3390/plants11111480

**Published:** 2022-05-31

**Authors:** Donatas Naugžemys, Jolanta Patamsytė, Silva Žilinskaitė, Yoichiro Hoshino, Audrius Skridaila, Donatas Žvingila

**Affiliations:** 1 Botanical Garden, University of Vilnius, Kairėnų Str. 43, 10239 Vilnius, Lithuania; genetikas@gmail.com (D.N.); silva.zilinskaite@bs.vu.lt (S.Ž.); audrius.skridaila@bs.vu.lt (A.S.); 2 Institute of Biosciences, Life Sciences Center, University of Vilnius, Saulėtekio Av. 7, 10257 Vilnius, Lithuania; jolanta.patamsyte@gf.vu.lt; 3 Field Science Center for Northern Biosphere, Hokkaido University, Sapporo 060-0811, Japan; hoshino@fsc.hokudai.ac.jp

**Keywords:** *Lonicera*, population genetic structure, polymorphism, molecular markers, speciation

## Abstract

Blue honeysuckle (*Lonicera caerulea* L.) is a promising berry crop producing edible early-ripening berries with a valuable chemical composition. We evaluated the genetic diversity of native *L. caerulea* populations from the western (Baltic states) and eastern (the Russian Far East and Japan) edges of the Eurasian range using inter-simple sequence repeat (ISSR) and chloroplast DNA (*psb*A-*trn*H and *trn*L-*trn*F) markers. The genetic relationships of populations and genotypes were analyzed using principal coordinate and cluster analyses (neighbor joining and Bayesian clustering). Sampling was carried out in two disjunct areas of this circumpolar species and the analyses showed clustering of individuals and populations according to geographic origin. The analysis of genetic structure based on ISSR markers showed that the studied populations of *L. caerulea* were highly differentiated. However, sequence analysis of two chloroplast DNA (cpDNA) regions revealed no phylogeographic structure among the populations. We also found that the eastern populations of blue honeysuckle had significantly greater genetic diversity parameters than the populations from the Baltic region. This finding correlates with the endangered status of blue honeysuckle in the Baltic states.

## 1. Introduction

Blue honeysuckle, *Lonicera caerulea* L. s.l. (Caprifoliaceae, Caeruleae), is a highly polymorphic species native to the Northern Hemisphere that exhibits a large range from eastern Scandinavia to Kamchatka in Eurasia and is widespread in North America [1,2]. It is a medium-sized perennial shrub that produces blue edible berries. The economic potential of this species has not yet been sufficiently exploited, although blue honeysuckle has been intensively studied in recent years, and opportunities for its wider use to meet human needs are being sought. Blue honeysuckle attracted the interest of breeders as recently as the middle of the last century [1,3]. Currently, blue honeysuckle is considered a promising berry crop with very strong advantages: stable annual yield, extreme winter hardiness, early fruiting, and good fruit biochemical properties [1,3,4,5]. The berries of blue honeysuckle contain vitamin C and many phenols, flavonoids, anthocyanins, and other phytochemicals that determine their antibacterial, antioxidant, and antidiabetic features [6,7,8]. The cultivation areas of cultivars of this species are constantly growing, and since the end of 2018, these berries have been approved for marketing in the European Union [8,9].

An understanding of the genetic structure of natural and cultivated populations of this widespread polymorphic species would help identify the optimal selective direction for obtaining new stable productive forms and varieties [10]. On the other hand, progress in honeysuckle breeding and improvement of existing cultivars can be made by crossing geographically and genetically distant genotypes of *L. caerulea* [1,4] or perhaps through interploidy crosses [11].

Phytogeographic studies based on chloroplast DNA (cpDNA) region and internal transcribed spacer (ITS) sequencing suggest that the clade of Caprifoliaceae, which includes the genus *Lonicera*, is native to Asia, where species diversification has taken place. Since diversification, there have apparently been multiple dispersal events to Europe and North America [12]. The honeysuckles (genus *Lonicera*) are thought to have originated and evolved in present-day China, as plants from China are characterized by all the characteristics of plants from Central Asia, Transbaikalia, and Japan [13]. Blue honeysuckle grows in the wild mainly in Russia, Japan, China, Canada, and the USA. According to Plekhanova [1], the richest genetic diversity is concentrated in northeastern Russia. In contrast, at the western border of the range, the Baltic populations of *L. caerulea* are rare and small. In the Baltics, as a result of anthropogenic activities related to the development of forests and drainage of bogs, this species is threatened with extinction [14,15]. At the western periphery of the range, *L. caerulea* grows in the wet forests of Latvia (Tukums region: Lake Slokas, Lake Kanieris, and the Slocene river valley) and Estonia (Raple district, Järvakandi). In the central part of Russia, populations of *L. caerulea* are also rare, and the species is considered endangered [15].

Various studies conducted in the past suggest that populations at the edge (periphery) of the species range often exist under suboptimal conditions [16,17]. A lack of suitable habitat or disturbance may lead to small population sizes, which may cause increased inbreeding and genetic drift. These factors can lead to reduced levels of genetic diversity and loss of alleles important for adaptation [17,18]. As a result, populations may lose the ability to adapt to environmental change caused by global warming and direct human impact.

Natural polymorphisms of blue honeysuckle are characterized by a high level of individual (intrapopulation) variability, which is shown by a number of morphological features, such as shoot color and length, leaf shape, tube, and pedicel lengths, corolla color, and fruit shape [10]. A comparison of geographical populations of blue honeysuckle revealed a pattern of adaptive variability not only in morphological characters but also in the features of morphogenesis, which is associated with climatic zones and high-mountain regions with altitudinal zonation (from forest to alpine belts) [10,13].

Although there have been many taxonomic, biological, and biochemical studies on honeysuckle, rather few reports have been published on the wild population genetic structure of *L. caerulea* assessed using molecular markers. Holubec et al. [4] studied genotypes from Sakhalin and Kamchatka in terms of morphological and genetic variability, taxonomic reliance, and genetic resources for breeding. Analyses based on amplified fragment length polymorphism (AFLP) markers revealed significant differences between blue honeysuckle populations from Sakhalin and Kamchatka and supported the results of phenotyping carried out using morphological characters. In a recent study by Smolik et al. [9], 24 accessions with different origins from the Polish, Ukrainian, Estonian, and Russian collections were analyzed according to their genotypic variability, population structure, and genetic relationships. The authors revealed genetic differences between cultivated and wild genotypes. Native genotypes from the Alps and Russian Far East formed separate clades on dendrograms constructed on the basis of ISSR, RAPD, and R-ISSR markers. Previous molecular diversity studies of blue honeysuckle were conducted mainly on germplasm collections based on material from Russia [2,19,20,21,22]. Dominant molecular markers (random amplified polymorphic DNA (RAPD) and ISSR markers) were used to assess genetic diversity in these studies, revealing the potential of genetic collections to serve as valuable resources for breeding blue honeysuckle cultivars.

In this study, we evaluated the polymorphism of ISSR and cpDNA markers in blue honeysuckle populations. ISSRs are universal DNA markers that, if carefully optimized, can be used for population studies in any species [23,24]. Markers based on cpDNA sequences are accurate molecular instruments suitable for species identification and phylogenetic assessment [25]. Here, we studied the genetic diversity of blue honeysuckle populations from western (Baltics states) and eastern (the Russian Far East and Japan) regions of the Eurasian range using ISSR and cpDNA (*psb*A-*trn*H and *trn*L-*trn*F) markers and tested the hypothesis that the genetic diversity of western populations experiencing suboptimal conditions is lower than that of populations in the areas of origin and that are widespread.

## 2. Materials and Methods

### 2.1. Plant Material

**Figure 1:**
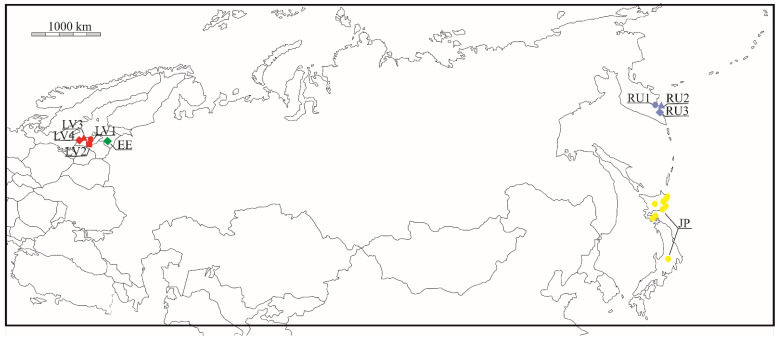
Map of the locations of the studied *Lonicera caerulea* populations. Different colors indicate different countries of origin of the populations. Red–Latvia, green–Estonia, blue–Russia and yellow–Japan. The population codes are explained in Table 1.

A total of 140 individuals of blue honeysuckle were sampled, representing 9 native populations: Japan (Hokkaido and Honshu Islands)—23, Kemeri (Latvia)—12, Kandava (Latvia)—12, Mikeltornis (Latvia)—11, Ventspils (Latvia)—11, Kalli (Estonia)—14, Elizovo (Kamchatka, Russia)—22, Petropalovsk-Kamchatsky 1 (Kamchatka, Russia)—17, and Petropalovsk-Kamchatsky 2 (Kamchatka, Russia)—19 (Table 1; Figure 1).

Samples were taken at random from plants separated by a distance of at least 10 m, except for the Japanese honeysuckle population, where samples were collected from different parts of Hokkaido (19 samples) and Honshu (4 samples) (see [26]).

**Table 1:** Sampling information, code and habitat characteristics of *Lonicera caerulea* populations.

Sample Number	Site	Code	Habitat	Coordinates (Latitude N, Longitude E)	Altitude, m	Climate Zone ^1^
JAPAN						
1	Taiki, Hokkaido	JP	Alder forest, *Quercus crispula* and *Q. dentata* forest	42.31–42.33, 143.28–143.30	0–70	Dfb
2–3	Yokotsu-dake, Hokkaido	JP	Rocky area around mountaintop	41.56, 140.46	1120–1150	Dfb
4	Taisetsu, Hokkaido	JP	Around mountainous mire	43.36–43.89, 142.53	1700–1800	Dfb
5	Betsukai, Hokkaido	JP	Area drained by agricultural development in Fuhren mire	43.16, 145.11	20	Dfb
6	Bekanbe, Hokkaido	JP	Alder forest	43.10, 144.51	0–10	Dfb
7–14	Kiritappu, Hokkaido	JP	Intermediate moor	43.02–43.08, 145.01–145.06	0–10	Dfb
15–18	Nikko, Tochigi, Honshu	JP	Area surrounding Senjogahara mire	36.46, 139.26	1390–1400	Dfa
19	Tomakomai, Hokkaido	JP	Area drained by industrial development	No data	No data	Dfb
20	Yufutsu, Hokkaido	JP	Area drained by industrial development	42.40,141.45	No data	Dfb
21–23	Apoi-dake, Hokkaido	JP	Mountain area	42.06, 143.01	600	Dfb
LATVIA						
24–35	Kemeri	LV1	Fen with birch and willow	56.97, 23.54	0–79	Dfb
36–47	Kandava	LV2	Abava river valley, open place, shrubs with *Pentaphylloides fruticosa*	57.02, 22.78	38–41	Dfb
48–58	Mikeltornis	LV3	Baltic Sea coastal pinewood	57.60, 21.97	8	Dfb
59–69	Venspils	LV4	Baltic Sea coastal pinewood, dunes	57.45, 21.60	8	Dfb
ESTONIA						
70–83	Kalli	EE	Fen with alder and birch	58.50, 24.09	22	Dfb
RUSSIA						
84–104	Elizovo	RU1	Swamp, peat soils, bumps, puddles Mossy marsh with thickets of alder and willow	53.24 158.40	21	Dfc
105–121	Petropavlovsk-Kamchatsky 1	RU2	Krasnaya Sopka, uphill, southeastern slope. Dry soils, peat, volcanic slag. Forest with birch, alder, willow, wild rose	52.99, 158.67	200	Dfc
122–140	Petropavlovsk-Kamchatsky 2	RU3	Zerkalnaya Sopka, on the slope of the hill. Forest with birch, wild rose, alder, rowan	53.04, 158.67	147	Dfc

^1^ according to the Köppen–Geiger climate classification [27]; Dfa, cold, no dry season, hot summer; Dfb, cold, no dry season, warm summer; Dfc, cold, no dry season, cold summer.

### 2.2. Genomic DNA Extraction and ISSR-PCR Analysis

Fresh and healthy leaves were collected from plants growing in natural habitats. The leaves were dried in bags with silica gel. DNA was extracted using a modified CTAB method [28] and adjusted for a small amount of plant material. The quantity and quality of DNA were measured by a NanoDrop 2000 spectrophotometer (Thermo Scientific, Wilmington, DE, USA) and by agarose gel electrophoresis. ISSR-PCR was carried out as described earlier by Butkuvienė et al. [29]. Briefly, blue honeysuckle DNA amplification was carried out in 20 μL reaction mixtures containing the following components: 2 μL of 10 × PCR buffer, 300 μM MgCl_2_, 200 μM dNTPs, 20 ng of template DNA, each primer at 0.4 μM and 1 U of Taq polymerase (Thermo Fisher Scientific/Baltics, Vilnius, Lithuania). ISSR-PCR was carried out in a Mastercycler ep gradient (Eppendorf AG, Hamburg, Germany) as follows: 94 °C for 7 min; 32 cycles of 94 °C for 30 s, the temperature for each primer (39 °C for ISSR I-28 and ISSR I-32, 42 °C for ISSR I-39a, 44 °C for ISSR O, 45 °C for ISSR I-34, 47 °C for ISSR I-50a and ISSR E, 49 °C for ARCADE 3 and ARCADE 4, and 60 °C for ISSR G) for 45 s, and 72 °C for 2 min; and ending with 72 °C for 8 min. Of the 23 tested ISSR primers, 10 were chosen for further study, all reactions were carried out at least twice in separate experiments, and reliable bands were scored. A negative control (water instead of DNA) was included in each experiment. Amplified DNA fragments were run in 1.5% agarose gels and 0.5 × TBE buffer at a constant voltage of 3.0 V/cm for 5 h. The gels were stained with ethidium bromide and examined under UV light using a BioDocAnalyse System (Biometra, Göttingen, Germany). DNA fragment size adjustment among specimens from different populations and the evaluation of the genotyping error rate were performed as described by Patamsytė et al. [30]. The observed genotyping error was 1.0%. GeneRuler^TM^ DNA Ladder Mix (Thermo Fisher Scientific/Baltics, Vilnius, Lithuania) was used as the DNA fragment size standard.

### 2.3. Chloroplast DNA Analysis

Two cpDNA regions (*psb*A-*trn*H and *trn*L-*trn*F) were analyzed by choosing 3–10 randomly selected individuals from each population (63 individuals in total) (Table S1). Amplification of the *psb*A-*trn*H and *trn*L-*trn*F regions was performed using universal primers previously described by Shaw et al. [31]. DNA sequences of individuals representing different populations were deposited in GenBank under accession numbers ON212105–ON212166 for *psb*A-*trn*H and ON212167–ON212228 for *trn*L-*trn*F.

### 2.4. Data Analysis

Scored ISSR-PCR products (DNA bands) were recorded and used to construct a 0/1 (binary) data matrix, which was used for further data analyses. Because blue honeysuckle from the studied populations is tetraploid (2n = 4x = 36), only genotype 0000 was scored as 0, whereas all other genotypes at a particular locus (1000, 1100, 1110, and 1111) were indistinguishable on the gel due to the dominant nature of ISSR markers and were considered as 1. The number of alleles per locus (N_a_) and the number of effective alleles (N_e_) were calculated using GenAlEx v 6.5 [32]. Additionally, the rarefaction procedure was applied for estimations to compensate for uneven population sample sizes, and the number of molecular phenotypes (band richness—Br) was calculated per locus in each population using AFLPDIV [33]. The rarefaction procedure was also used to compute the percentage of polymorphic loci (PLP) at the 5% level. After rarefaction, the standardized sample size was 11 genotypes. Shannon’s information index (I) was calculated using POPGEN 1.32 software [34]. The calculation of expected heterozygosity (Hj) within populations was carried out with AFLP-SURV [35]. We assessed whether it was possible to apply the isolation-by-distance (IBD) model to describe the pattern of genetic diversity variation among the studied populations. For this purpose, the association between genetic (Φ_PT_) and geographic distance matrices were examined with a Mantel test using GenAlEx v 6.5 [32]. A total of 999 permutations were performed for significance testing.

On the basis of binary data, pairwise genetic distances between individuals were calculated and used in the MEGA 11 [36] software package for neighbor-joining (NJ) [37] dendrogram construction to reveal genetic relationships among individuals and populations. The support for dendrogram branches was evaluated using 1000 bootstrap replications. Principal coordinate analysis (PCoA) was performed using GenAlEx v.6.5 to assess the genetic relationships among populations from western and eastern Eurasian regions. Nei’s average number of pairwise differences in nine *L. caerulea* populations was determined by Arlequin 3.5.2.2 [38].

The genetic differentiation of populations was ascertained according to Fst values using AFLP-SURV v.1.0 [35] based on 10,000 random data permutations. Three-level analysis of molecular variance (AMOVA) performed in GenAlEx v.6.5 [32] was used to ascertain the partitioning of genetic diversity among regions, among populations within regions, and among individuals within populations. To determine and compare the genetic structures of honeysuckle populations and the distribution of individuals by clusters (K), the STRUCTURE program version 2.3.4 was used [39,40]. To quantify the number of clusters, ten independent runs of K (K = 1–10) were performed with an admixture model, with 100,000 burn-in iterations and 500,000 Markov chain Monte Carlo iterations. The most likely K was identified with the ΔK method of Evanno et al. [41]. The STRUCTURE result files were processed in STRUCTURE HARVESTER, and the output was visualized using DISTRUCT v1.1 [42].

Sequencing data of amplified *psb*A-*trn*H and *trn*L-*trn*F regions were evaluated with MEGA 11 [36]. Genetic diversity parameters (Shannon’s information index (I), number of alleles per locus (Na), expected heterozygosity (Hj), percentage of polymorphic loci at the 5% level (PLP), and band richness (Br)) were compared between populations from the western and eastern Eurasian regions using the Mann–Whitney U test in IBM SPSS Statistics v.23 for Windows.

## 3. Results

ISSR analysis was carried out on 140 individuals from nine populations of *L. caerulea*. A total of 181 scorable bands (loci) were generated for *L. caerulea* using ten ISSR primers. The percentage of polymorphic bands ranged from 20.4% (37 polymorphic loci, LV4 population) to 53.6% (97 polymorphic loci, JP).

The calculations revealed that the estimates of observed alleles (N_a_) and effective alleles (N_e_) varied between 0.983 and 1.425 (LV3-JP) and 1.123 and 1.348 (LV2-JP), respectively (Table 2).

The average polymorphism per population was 27.62 ± 3.42 (mean ± SE). When the number of individuals per population was rarefied to N = 11, the proportion of polymorphic loci (PLP 5%) varied from 0.204 (LV4) to 0.558 (JP) (mean PLP 5% = 0.283 ± 0.037). The band richness (Br) was also adjusted according to the smallest population size (11 individuals) and ranged from 1.204 (LV4) to 1.512 (JP). The Shannon index (I) ranged from 0.108 (LV2) to 0.3 (JP), with an average of 0.153 ± 0.02. The expected heterozygosity (Hj) varied from 0.076 (LV2) to 0.199 (JP), with an average of 0.104 ± 0.026. Only five population-specific bands were identified. One private band was detected in the RU2 population, and four private bands were identified in the JP population. A Mantel test showed a correlation (R^2^ = 0.479; *p* = 0.01) between genetic and geographic distances among populations.

**Figure 2:**
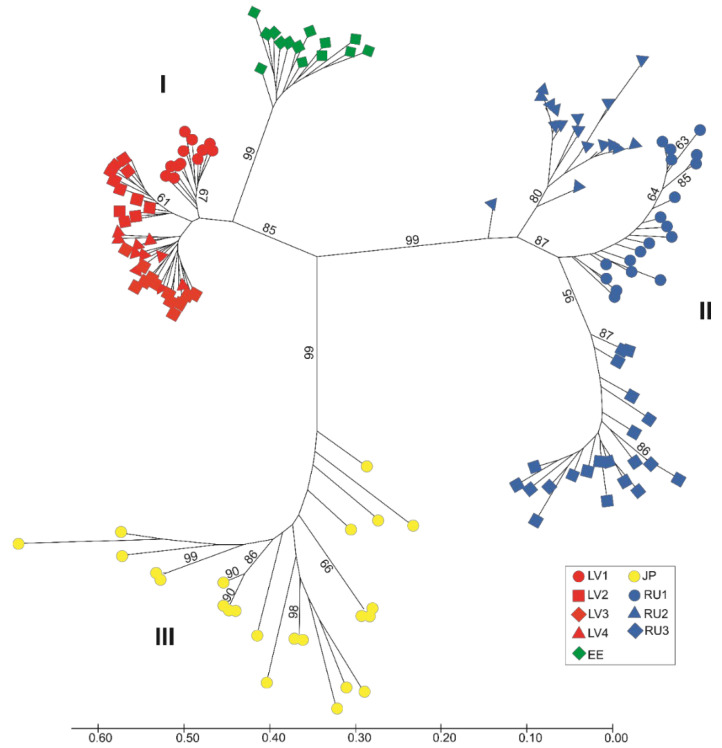
NJ dendrogram of the genetic relationships between nine *Lonicera caerulea* populations. Bootstrap values (%) obtained after 1000 iterations are shown as numbers above the branches. Only values above 60% are shown. The scale at the bottom shows the genetic distance. I, II, and III—clusters.

The genetic relationships of *L. caerulea* populations and genotypes included in our study were analyzed using NJ cluster analysis and PCoA. The NJ cluster analysis grouped all individuals into three main clusters with high bootstrap support (Figure 2).

Populations, in general, were grouped according to geographic origin. The first cluster included samples from the LV1, LV2, LV4, and LV3 populations. Cluster analysis showed that the LV3 and LV4 populations were closely related, and their individuals were mixed in the dendrogram. The EE population was also placed in the first cluster but formed a separate subcluster. All populations in the first cluster were from the Baltic region. The second cluster included populations from the Russian Far East (RU2, RU1, and RU3). The third cluster contained honeysuckles from Japan. Samples collected in mainland Japan (Nikko, Tochigi) were grouped within the Hokkaido Island samples in the dendrogram (not specified).

**Figure 3:**
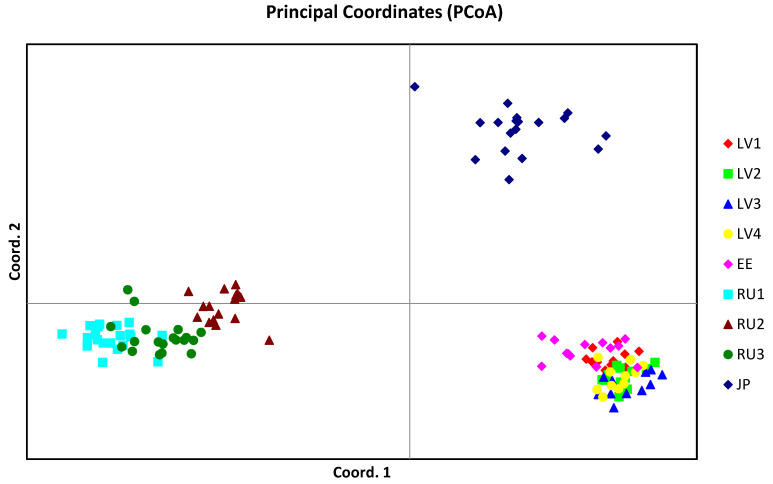
PCoA of *Lonicera caerulea* individuals from nine populations based on Nei’s genetic distance. Two coordinates are shown, which explained 75.14% of the variation.

In the PCoA, the first three axes explained most of the variation (84.54%). PC1 explained 48.44%, PC2 explained 26.7% and PC3 explained 9.4% of the variability. The results of the analysis revealed three clusters of individuals and populations grouped according to geographic origin (Figure 3).

The two-dimensional scatter plot showed the most compact distribution pattern and overlap for Baltic region populations. The graph also revealed the grouping of Russian Far East populations with evident overlap between the RU1 and RU3 populations.

**Figure 4:**
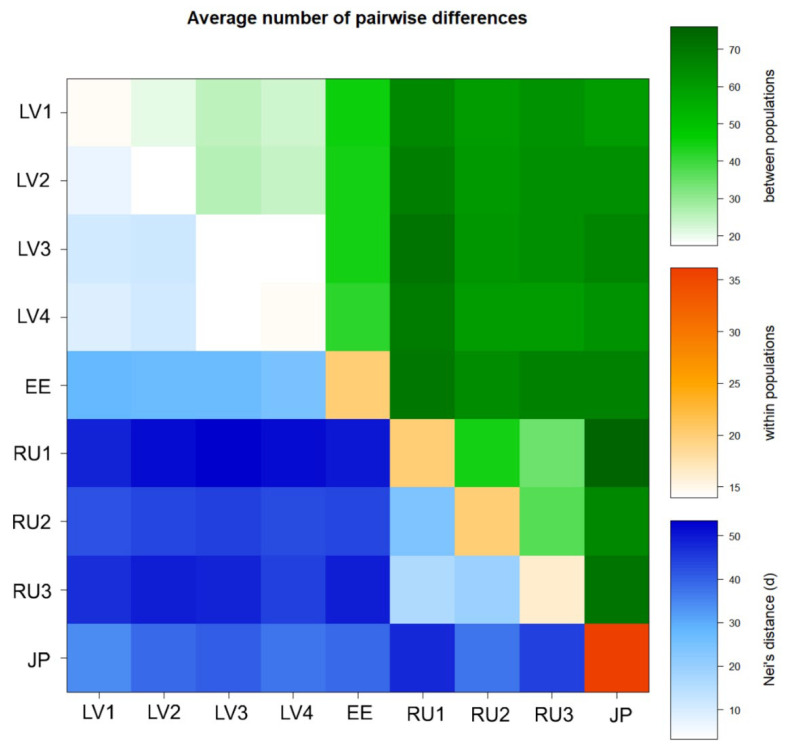
Pattern of the average number of pairwise differences between nine populations of *Lonicera caerulea*. Blue colors under the diagonal indicate Nei’s distance between populations; orange colors on the diagonal indicate differences within populations; green colors above the diagonal indicate differences between populations.

The genetic differentiation between the studied honeysuckle populations was high (Fst = 0.648 ± 0.022). We also evaluated population structure with hierarchical AMOVA to assess how genetic variability is partitioned among regions and populations and within populations. The results revealed similar proportions of variability among regions (30%, *p* = 0.001), among populations within regions (39%, *p* = 0.001) and within populations (31%, *p* = 0.001). The pairwise divergence between the studied *L. caerulea* populations and the variation within populations is visualized in Figure 4.

The generated pattern of pairwise differences indicated that populations from eastern regions of Eurasia were more diverse than western populations. The Japanese honeysuckle population (JP) possessed the highest within-population diversity.

**Figure 5:**
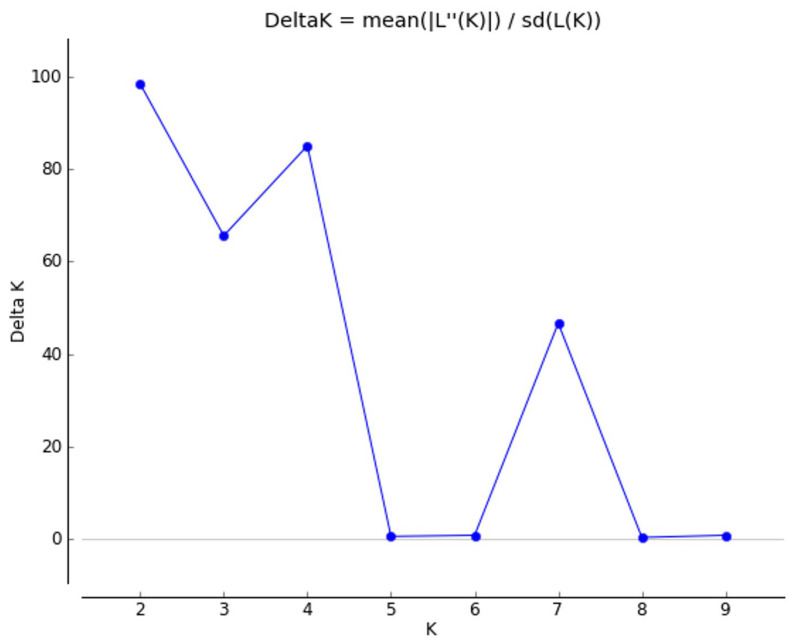
Application of the Evanno et al. [41] approach to identify the true cluster number (K) from STRUCTURE analyses. The graph shows peaks of ΔK values at K = 2, K = 4, and K = 7.

In assessing population genetic structure extrapolated using STRUCTURE software, we observed a major peak of real structure at K = 2 (Figure 5).

**Figure 6:**
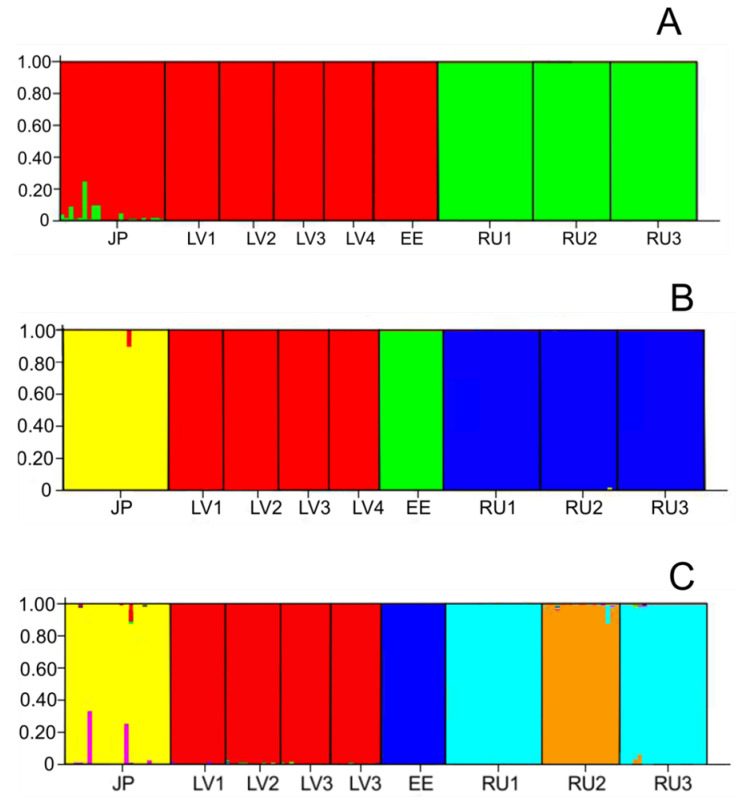
STRUCTURE graphs showing Bayesian assignments of *Lonicera caerulea* individuals to K genetic clusters. (**A**) K = 2, (**B**) K = 4, and (**C**) K = 7. The nine populations are separated by black vertical lines. The population codes are the same as those described in Table 1.

In general, the most likely pattern of clustering separated the populations according to geographic origin, with the exception of the Japanese population (Figure 6).

All western populations and the population from Japan were grouped into the red cluster, while populations from Kamchatka (RU1, RU2, and RU3) were assigned to the green cluster (Figure 6A). Some individuals from Japan showed admixture. Peaks were also observed at K = 4 and at K = 7 (Figure 5). In the case of K = 4, populations were clustered similarly to those in the previous grouping (K = 2), except that the JP population and EE population were separated into single clusters, yellow and green, respectively (Figure 6B). The red cluster encompassed all four Latvian populations, and the blue cluster included all three Russian populations. At K = 7, heterogeneity was revealed among Russian populations by grouping the RU2 population into separate clusters (Figure 6C). Some individuals from Hokkaido (JP population) were admixed and had genomes originating from different gene pools. They were assigned to the yellow cluster with a lower probability (*q* < 0.8) than other genotypes of the same population.

No polymorphism was detected in the psbA-*trn*H and *trn*L-*trn*F sequences from 63 samples of different populations. The lengths of the *psb*A-*trn*H and *trn*L-*trn*F regions of *L. caerulea* were 559 nt and 1015 nt, respectively.

## 4. Discussion

### 4.1. Genetic Diversity

Different levels of DNA polymorphism were revealed in the nine studied blue honeysuckle populations. The highest polymorphism (53.6%) was detected in plants from the JP population, which included samples from Hokkaido Island and a few samples from Tochigi Prefecture (Honshu Island). Populations from Kamchatka (RU1, RU2, and RU3) showed a moderate frequency of polymorphic loci (49.7%). The lowest level of polymorphism was observed in populations from Latvia (37.6%). The lowest values of Na, Ne, I, Hj, and Br were also observed for LV populations. Although the EE population showed greater genetic diversity, in general, the populations in the western part of the range were less genetically diverse than those in the eastern part. According to the Mann–Whitney test, populations from the eastern part of the blue honeysuckle range had significantly greater I (*U* = 1.00, Z = −2.205, *p* < 0.05), Br (*U* = 1.00, Z = −2.205, *p* < 0.05), and PLP 5% (*U* = 1.00, Z = −2.214, *p* < 0.05) values than populations from the Baltic region. *L. caerulea* is a fairly widespread species on the Kamchatka Peninsula and usually forms large populations [4], while in the Baltic region, it is an endangered species with only a few fragmented populations known. In addition to the negative effects of anthropogenic factors, climate warming with frequent thaws in winter can also have a negative impact on the populations of the Baltic region. Nevertheless, the populations of Kamchatka are also facing increasing anthropogenic impacts, leading to fragmentation and overexploitation of some populations [4]. By means of the AFLP assay, higher values of population polymorphism in Kamchatka (83.6%) and Sakhalin (69.9%) were detected by previous authors. However, the collection of samples was more geographically scattered than in our study.

### 4.2. Genetic Structure of Populations

Our study shows that populations from different edges of the Eurasian blue honeysuckle range are highly differentiated. Similar patterns of genetic structure were obtained using different statistics (PCoA, NJ, and AMOVA). For example, PCoA revealed a divergence between remote populations and reliably subdivided all individuals into three groups. NJ analysis also showed three main clusters. Additionally, AMOVA showed a strong genetic structure in blue honeysuckle. Based on molecular and morphological data analyses, Holubec et al. [4] revealed a divergence (F_ST_ = 0.19) between native blue honeysuckle populations from Sakhalin and Kamchatka. Polymorphism of AFLP markers allowed these populations to be separated into different clusters in a PCoA plot and an NJ dendrogram. In general, the genetic structure of plant populations indicates the interplay among various evolutionary factors (genetic drift, mutations, natural selection, habitat fragmentation and isolation, mating systems, gene flow, and migration) in the context of many years of species evolution [43]. The dispersed distribution of *L. caerulea* across the very large circumpolar range of the Northern Hemisphere implies such complex evolutionary processes. Our results indicate that most of the genetic diversity occurs among populations. High genetic differentiation among populations is usually not characteristic of perennial outcrossing plant species [44,45]. However, some of the abovementioned factors associated with the evolution and ecology of particular species can cause deviations from typical patterns of genetic variability [43,44,46,47,48]. For example, Shrestha et al. [44] explained high differentiation among populations of the outcrosser *Acacia raddiana* by genetic drift and local adaptation. Vashishtha et al. [47] detected high genetic differentiation among populations of the tropical tree *Butea monosperma* independent of the marker assay used, i.e., RAPD, ISSR, or sequence-related amplified polymorphism (SRAP). The authors of this study noticed that substantial population divergence may be determined by a low level of gene flow among the studied populations due to geographic distance, heterogeneity of ecological conditions, and possibly mixed breeding systems. The strong population differentiation of *Juniperus communis* in Lithuania (G_ST_ = 0.491) was dependent on habitat fragmentation and heterogeneity [49]. Low gene flow among the studied blue honeysuckle populations (indicated by high Fst) may be responsible for the increased genetic heterogeneity among them [50]. The geographic distance among populations and ecological differences in the habitats of some *L. caerulea* populations may be the causative factors of low gene flow among them. The western and eastern populations included in our study are separated by large distances. Moreover, honeysuckle populations in Japan (Hokkaido and Honshu Islands) are separated from continental populations located on the Kamchatka Peninsula. The Mantel test showed that geographic distance is important for population divergence. Our results indicate significant IBD (*p* = 0.01). In addition, differences in the ecological environments of habitats among populations may also be important for population differentiation [4,47,51,52]. Anthropogenic activities and perhaps climatic fluctuations caused fragmentation of *L. caerulea* populations (especially in the Baltic region). In such situations, populations may be impacted by genetic drift, which increases population differences and decreases variation within populations. The divergence between the studied populations is caused mainly by differences in allele frequencies. Only two populations (JP and RU1) possessed a few private bands. Three western populations (LV3, LV4, and EE) also did not show bands of low frequency (≤25%). The remaining populations had only one or two bands in this frequency range. Another interesting aspect of the obtained results is that the Japanese population grouped separately from the eastern populations (according to NJ and PCoA results; Figure 2 and Figure 3) and, according to Bayesian cluster analysis, showed some similarity to the Baltic populations (Figure 6A). Geographically, the Kamchatka (Russia) populations (RU1, RU2, and RU3) are closer to the JP population than populations from the Baltic region. This discrepancy could be the result of adaptation to local environmental conditions [44,53,54]. For example, Kamchatka populations experience a more severe climate with colder winters and shorter summers than the other studied populations. Notably, according to the Köppen–Geiger climate classification, the Baltic region and the northern part of Japan (including Hokkaido) are assigned to the Dfb climate subtype, and the northern part of Honshu is assigned to Dfa, while the climate of the Kamchatka Peninsula is assigned to the Dfc subtype [27]. The studies of various authors [55,56,57,58] have revealed great morphological diversity of honeysuckle populations situated in different parts of the range, which also resulted in an ambiguous taxonomic assessment of blue honeysuckle. Recently, however, there has been a return to Rehder’s [59] idea [4,60,61,62] that blue honeysuckle is a polymorphic species with complex intraspecific taxonomy and that the morphological differences found among isolated populations are likely the result of local adaptation. This point of view is corroborated by our finding from a cpDNA analysis, which showed no phylogeographic structure among the studied populations. No polymorphism was detected in the two cpDNA regions frequently used in barcoding analyses [31,63,64]. However, we cannot exclude the occurrence of microevolutionary processes at the species level. Authors currently recognize that within *L. caerulea* there exists a taxonomically complex intraspecific structure, which manifests itself in the existence of morphological, physiological, and genetic differences [4,60,65,66]. Thus, the large degree of population differentiation that we found based on ISSR markers may also reflect the processes of intraspecific divergence resulting from the adaptive evolution of populations [51]. Differences in the genetic structure of populations evaluated using ISSR and cpDNA markers could also be explained by different rates of evolution of nuclear and chloroplast genomes, as it is known that the nuclear genome evolves faster than the chloroplast genome [25]. ISSR markers are associated with microsatellites. The mutability of microsatellite loci is significantly higher than that of most other types of DNA sequences [67]. Recently, Garshasbi et al. [68] revealed 75% of genetic variability among seven *Lonicera* species in Iran on the basis of ISSR polymorphisms. In this regard, the parameters of population genetic differentiation established by us indicate the presence of pronounced genetic structure within *L. caerulea*, which suggests that speciation processes are taking place.

## 5. Conclusions

Our study of DNA polymorphisms in populations confirmed the opinion of other authors that geographically separated blue honeysuckle populations are genetically divergent. In this study, this was determined at the level of noncoding DNA (ISSRs). The revealed high population differentiation reflects the complex intraspecific taxonomic structure of this honeysuckle species. On the other hand, the lack of polymorphism at the cpDNA level indicates the integrity of diverged populations or subspecies of one species. Populations on the western edge of the species range (especially in Latvia) are genetically less diverse than blue honeysuckle populations on the opposite edge of Eurasia. This correlates with their current endangered status in the Baltic states. As economic interest in *L. cerulea* continues to increase, studies on the genetic structure of blue honeysuckle will provide information about processes occurring in geographically isolated populations that are potential sources of genetic diversity and may contribute to the correct assessment of population divergence. More extensive studies of genomic polymorphisms are needed to develop a complete picture of the evolution of the species.

## Figures and Tables

**Table 2:** Genetic diversity parameters of the studied *Lonicera caerulea* populations by region based on ISSR markers.

Pop	N	PLP (5%) [11]	N_a_	N_e_	I	Hj	Br [11]
JP	23	0.558	1.425	1.348	0.300	0.199	1.512
LV1	12	0.232	1.022	1.137	0.119	0.079	1.226
LV2	12	0.210	0.989	1.123	0.108	0.076	1.206
LV3	11	0.210	0.983	1.136	0.114	0.078	1.210
LV4	11	0.204	0.989	1.134	0.111	0.079	1.204
EE	14	0.271	1.044	1.185	0.154	0.111	1.263
RU1	21	0.315	1.110	1.195	0.168	0.110	1.284
RU2	17	0.293	1.094	1.179	0.158	0.110	1.274
RU3	19	0.254	1.000	1.167	0.141	0.090	1.234
Eastern region (JP + RU1 + RU2 + RU3)							
Mean ± SE	80	0.355 ± 0.062	1.157 ± 0.093	1.222 ± 0.042	0.192 ± 0.036	0.127 ± 0.022	1.326 ± 0.056
Baltic region (LV1 + LV2 + LV3 + LV4 + EE)							
Mean ± SE	60	0.225 ± 0.014	1.006 ± 0.012	1.143 ± 0.011	0.121 ± 0.008	0.085 ± 0.008	1.222 ± 0.013
Overall							
Mean ± SE	140	0.283 ± 0.077	1.073 ± 0.047	1.178 ± 0.023	0.153 ± 0.020	0.104 ± 0.026	1.268 ± 0.068

N, number of plants studied per population; PLP (5%), percentage of polymorphic loci at the 5% level for population size rarefied to 11 individuals; N_a_, number of alleles per locus; N_e_, number of effective alleles; I, Shannon’s information index; Hj, expected heterozygosity; Br, band richness based on 11 individuals.

## Data Availability

The chloroplast DNA sequences are available on NCBI GenBank (accession numbers *psb*A-*trn*H: ON212105–ON212166; *trn*L-*trn*F: ON212167–ON212228).

## Supplementary Materials

The following supporting information can be downloaded at: https://www.mdpi.com/article/10.3390/plants11111480/s1; Table S1: *Lonicera caerulea* sample codes and NCBI GenBank accession numbers of corresponding *psb*A-*trn*H and *trn*L-*trn*F region sequences.
