# Supplementary material for: Genetic Structure of Native Blue Honeysuckle Populations in the Western and Eastern Eurasian Ranges
Source: Plants (Basel). 2022 May 31;11(11):1480. doi: 10.3390/plants11111480 (PMC9182990; doi:10.3390/plants11111480)
Supplement: Supplementary file 1 [file plants-11-01480-s001.zip › plants-1724002-supplementary.pdf]

Table S1. *Lonicera caerulea* sample codes and NCBI GenBank accession numbers of corresponding psbA-trnH and trnL-trnF region sequences.

Sample code	psbA-trnH	trnL-trnF
LV1-1	ON212105	ON212167
LV1-2	ON212106	ON212168
LV1-3	ON212107	ON212169
LV1-6	ON212108	ON212170
LV1-12	ON212109	ON212171
LV2-1	ON212110	ON212172
LV2-3	ON212111	ON212173
LV2-4	ON212112	ON212174
LV2-7	ON212113	ON212175
LV2-10	ON212114	ON212176
LV3-1	ON212115	ON212177
LV3-2	ON212116	ON212178
LV3-4	ON212117	ON212179
LV3-6	ON212118	ON212180
LV3-11	ON212119	ON212181
LV4-2	ON212120	ON212182
LV4-3	ON212121	ON212183
LV4-6	ON212122	ON212184
LV4-7	ON212123	ON212185
LV4-10	ON212124	ON212186
EE-1	ON212125	ON212187
EE-3	ON212126	ON212188
EE-4	ON212127	ON212189
EE-9	ON212128	ON212190
EE-10	ON212129	ON212191
EE-11	ON212130	ON212192
EE-14	ON212131	ON212193
RU1-1	ON212132	ON212194
RU1-3	ON212133	ON212195
RU1-4	ON212134	ON212196

RU1-5	ON212135	ON212197
RU1-10	ON212136	ON212198
RU1-13	ON212137	ON212199
RU1-14	ON212138	ON212200
RU1-15	ON212139	ON212201
RU1-18	ON212140	ON212202
RU1-20	ON212141	ON212203
RU2-1	ON212142	ON212204
RU2-3	ON212143	ON212205
RU2-7	ON212144	ON212206
RU2-9	ON212145	ON212207
RU2-13	ON212146	ON212208
RU2-16	ON212147	ON212209
RU2-17	ON212148	ON212210
RU3-1	ON212149	ON212211
RU3-2	ON212150	ON212212
RU3-4	ON212151	ON212213
RU3-6	ON212152	ON212214
RU3-10	ON212153	ON212215
RU3-11	ON212154	ON212216
RU3-12	ON212155	ON212217
RU3-18	ON212156	ON212218
JP-1-(TA)	ON212157	ON212219
JP-2-(YO-1)	ON212158	ON212220
JP-6-(BK)	ON212159	ON212221
JP-7-(KI-1)	ON212160	ON212222
JP-8-(KI-2)	ON212161	ON212223
JP-10-(KI-4)	ON212162	ON212224
JP-15-(NI-1)	ON212163	ON212225
JP-16-(NI-2)	ON212164	ON212226
JP-19-(TO)	ON212165	ON212227
JP-21-(AP-1)	ON212166	ON212228
